# Single-Application Biobrane Versus Nanocrystalline Silver Dressings for Bilateral Mid-Dermal Petrol Flash Burns: An Intra-Patient Split-Limb Case Report

**DOI:** 10.7759/cureus.116376

**Published:** 2026-09-16

**Authors:** Fergus J Walsh, Sophie G Rienecker, Lachlan Birrell

**Affiliations:** 1 General Surgery, Royal Darwin Hospital, Darwin, AUS

**Keywords:** biobrane, biosynthetic skin substitute, burn pain, case report, nanocrystalline silver, partial-thickness burn, split-limb comparison

## Abstract

Biobrane (Smith & Nephew, Hull, UK) is a biosynthetic bilaminate dressing used for superficial partial-thickness burns, where it provides single-application coverage until re-epithelialisation. Comparative data in adults with mixed-depth burns are limited, and within-patient comparisons are rare. We report a man in his early 50s with type 2 diabetes mellitus who sustained 21% total body surface area (TBSA) circumferential mixed superficial and mid-dermal burns to both lower limbs in a petrol flash explosion. At primary debridement the left limb was covered with a single application of Biobrane and the right with a silicone contact layer and nanocrystalline silver dressings. This created an unplanned intra-patient comparison in near-symmetrical wounds. The Biobrane limb required no further surgery and remained free of clinical infection. It healed by day 32. The silver-dressed limb required two further debridements. It developed a polymicrobial wound infection with non-multiresistant methicillin-resistant *Staphylococcus aureus* and *Pseudomonas aeruginosa*, needing systemic antibiotics. It healed later and was the site of persistent pain that continued after wound closure and required ongoing neuropathic analgesia. These complications drove a prolonged 23-day admission. The Biobrane limb outperformed the silver-dressed limb on every metric assessed: operations, infection, healing time, and pain. This case suggests that single-application biosynthetic coverage may offer analgesic and operative advantages in adult mixed-depth burns. It also highlights persistent pain as an underappreciated burden of silver-based dressing regimens.

## Introduction

Burn injuries are a common but under-appreciated form of trauma, with an estimated 11 million occurring annually worldwide and 180,000 of these proving fatal [1]. Most are caused by heat from hot liquids, solids, or fire, and the mechanism of injury shapes both the depth of the wound and the physiological response to it [1]. Partial-thickness burn care aims for rapid re-epithelialisation while minimising pain, dressing burden, and progression to grafting.

The central difficulty in meeting that aim is depth. Clinical assessment remains the most frequently used method of measuring burn depth but is accurate in only 60 to 75% of cases even when performed by an experienced burn surgeon, and it is markedly less accurate for burns of intermediate depth, which are common; overestimation of depth is the most frequent error [2]. Adult burns that may or may not heal by day 21 form a distinct indeterminate category in which management decisions are least secure [2]. Depth is also not fixed at the moment of injury. Tissue in the zone of stasis may progress to necrosis over the two weeks following injury, driven by prolonged inflammation, oedema with vascular congestion, microvascular occlusion, and bacterial infection [3]. The time taken to heal carries consequences beyond convenience: wounds healing within three weeks carry a low risk of hypertrophic scarring, whereas those taking longer carry a high risk, and delayed healing may itself result from infection or from impaired healing states such as diabetes mellitus [4].

Skin substitutes were developed to provide stable temporary coverage of such wounds. They can be divided broadly into biological and synthetic classes, differing in the presence of an extracellular matrix, the requirements of the wound bed, cost, and duration of adherence, and no single substitute is ideal for all indications [5]. Biobrane (Smith & Nephew, Hull, UK) is a bilaminate dressing of silicone and nylon mesh coated with porcine collagen peptides. It adheres to a clean superficial dermal bed and stays in situ until re-epithelialisation, avoiding repeated painful dressing changes [6-8]. Randomised trials have reported reduced pain, analgesic requirement, healing time, and length of stay versus 1% silver sulfadiazine in paediatric inpatients [6] and adult outpatients [7]. A Cochrane review found silver sulfadiazine was consistently associated with poorer healing than biosynthetic, silicone-coated, and silver dressings [9]. Broader use is limited by the need for a suitable wound bed, cost, and a perceived risk of subdressing infection [8,10,11].

Silver-impregnated dressings, including nanocrystalline silver, remain the default in many units for mixed-depth burns. They are valued for their antimicrobial profile and tolerance of exudative or indeterminate-depth wounds [12]. Meta-analysis of nanocrystalline silver against other silver delivery systems have reported fewer infections, fewer surgical procedures, and shorter hospital stays than silver sulfadiazine or silver nitrate [13]. Pain is the outcome on which the two strategies differed the most in principle. Burn pain comprises background, procedural, and breakthrough components, of which procedural pain arising from wound debridement and dressing changes was the major source of severe pain; patients did not habituate to it, it remained widely undertreated, and acute burn pain was linked in turn to persistent pain and psychological morbidity after wound closure [14]. Within-patient comparisons of the two dressing strategies in adults are rare, yet such a design is the one best able to hold constant the systemic factors that most strongly influence burn healing. We therefore report a split-limb comparison arising from routine care in a man with near-symmetrical bilateral lower limb petrol flash burns, in whom an unplanned difference in dressing allocation left every systemic variable shared between the two limbs. The aim of this report is to describe how the two strategies differed in operative burden, wound infection, healing time, and pain within a single patient, and to draw attention to persistent pain as an outcome that is rarely prespecified in comparative burn dressing research. Written informed consent was obtained for publication.

## Case presentation

A man in his early 50s with type 2 diabetes mellitus and obstructive sleep apnoea sustained flash flame burns when petrol vapour ignited at a service station, throwing him several metres. There was no loss of consciousness and no concern for airway or inhalational injury. He was intubated in the emergency department for analgesia and admitted to the intensive care unit of a tertiary referral burns service in northern Australia. Examination showed circumferential mixed superficial and mid-dermal burns to both lower limbs totalling 21% total body surface area (TBSA). There was no full-thickness component and compartments were soft throughout. Trauma computed tomography was unremarkable. Parkland-formula resuscitation was commenced. Initial over-resuscitation caused transient pulmonary oedema that resolved with diuresis, and he was extubated without complication.

On day one, he underwent operative scrubbing and debridement of both limbs, which established wound depth. The left limb burn was circumferential from ankle to the distal two-thirds of the leg. It extended anteriorly to the distal thigh and involved the dorsum of the foot and all toes. It was mixed superficial-dermal and mid-dermal (superficial and mid partial-thickness) throughout, with no deep-dermal or full-thickness component. It was covered with a single application of Biobrane. This allocation reflected Biobrane stock availability rather than clinical selection: both limbs were comparably suited to biosynthetic coverage. Only enough Biobrane was on hand at debridement to treat one limb, so the right received the department's standard silicone-silver regimen. The right limb burn was circumferential from ankle to knee, extending posteriorly to just above the knee, with a 0.5% TBSA patch on the dorsum of the foot and all toes. It was likewise predominantly superficial-dermal and mid-dermal. The only exception was a small deep-dermal (deep partial-thickness) component of approximately 0.5% TBSA at the malleoli and dorsum of the foot, later confirmed and debrided. It was dressed with a silicone contact layer (Mepitel; Molnlycke Health Care, Gothenburg, Sweden) and nanocrystalline silver (Acticoat; Smith & Nephew, Hull, UK).

The clinical course of the two limbs then diverged (Figures 1, 2).

**Figure 1:**
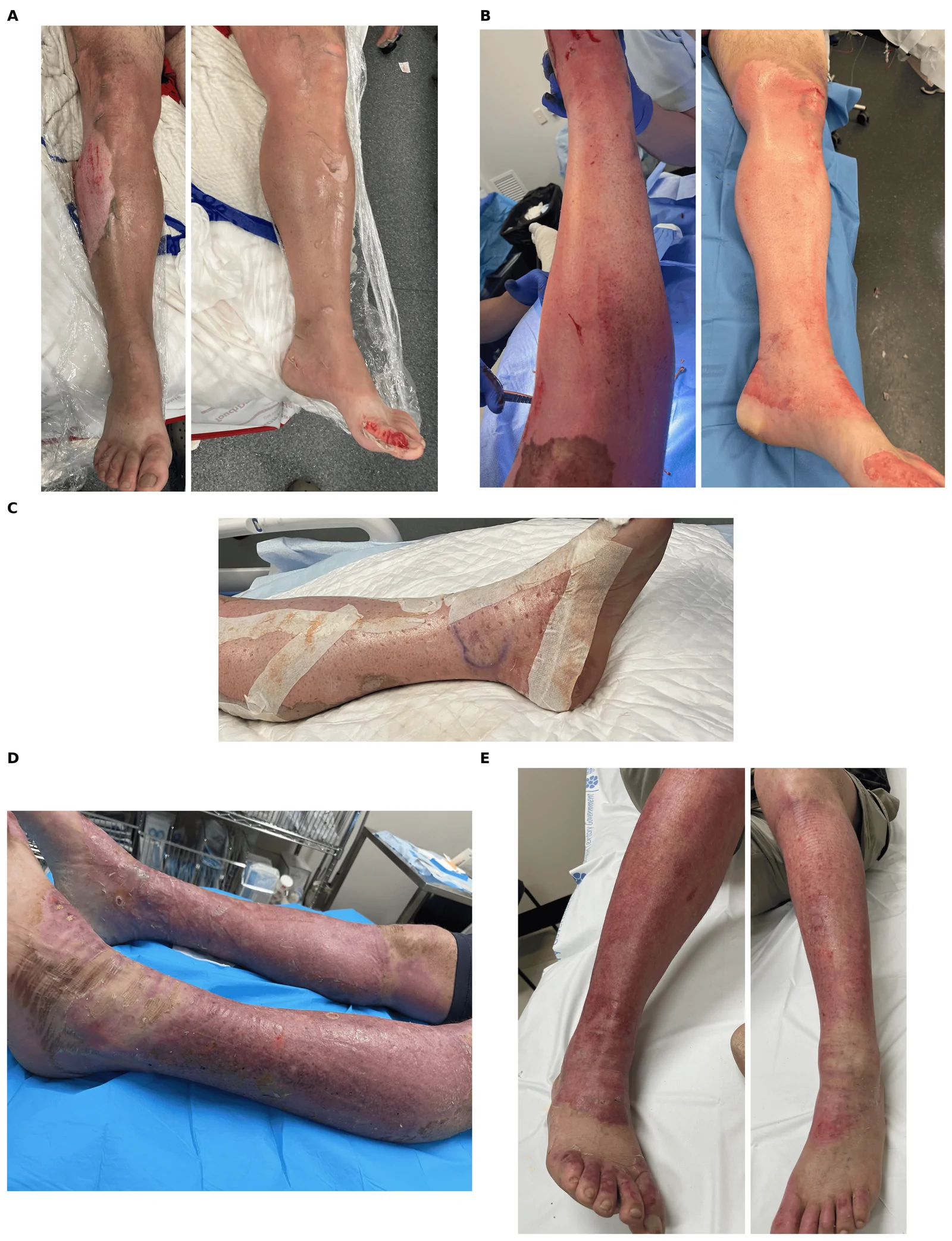
Clinical course of both lower limbs In paired panels the silver-dressed limb is on the left, the Biobrane limb on the right. (A) Day 0, before debridement; (B) Day 1, after debridement; (C) Day 3, Biobrane in situ; (D) Day 25; (E) Day 32.

The right limb returned to theatre on day four for debridement of pseudoeschar and redressing. It returned again on day nine. By then roughly 1.5% TBSA of mid-dermal wound remained, with a 0.5% sloughy deep-dermal area at the lateral ankle requiring debridement. A right lower limb swab grew non-multiresistant methicillin-resistant *Staphylococcus aureus* (nmMRSA) and *Pseudomonas aeruginosa*. This was treated with intravenous then oral clindamycin and ciprofloxacin plus topical silver sulfadiazine. The left (Biobrane) limb needed no further surgery and remained free of clinical infection.

Pain initially required a ketamine infusion, morphine patient-controlled analgesia, clonidine, tapentadol, and oxycodone, with inhaled nitrous oxide for dressing changes under acute pain service guidance. It was weaned to regular paracetamol, pregabalin, and amitriptyline with as-required oxycodone by discharge on day 23. At day 25, less than 1% TBSA remained open. The largest wound was a 5 x 4 cm area over the right lateral ankle. At day 29, the patient could not stand for more than three minutes because of pain in the right leg, despite roughly 98% re-epithelialisation. By day 32, the left leg had healed completely. The right was 99% healed with a residual lateral malleolar wound, and compression garments were fitted. A single 5 x 5 mm right malleolar wound persisted at day 39. Pain limiting standing and return to work remained confined to the right limb after wound closure and still required neuropathic agents. The Biobrane limb was pain-free.

**Figure 2:**
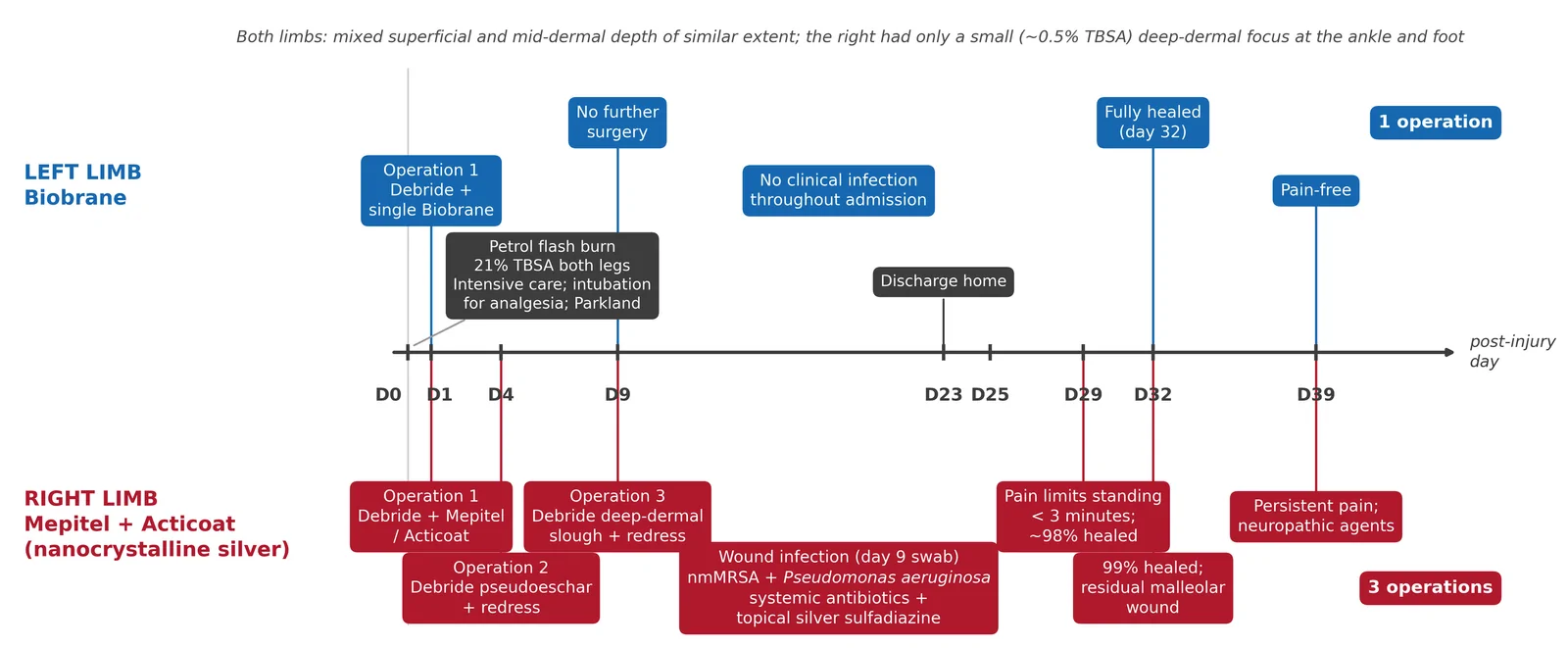
Split-limb clinical timeline The two lower limbs are plotted against post-injury day. The left limb (Biobrane) underwent a single operation, remained free of clinical infection, healed by day 32, and was pain-free. The right limb (silicone contact layer and nanocrystalline silver) underwent three operations, developed a polymicrobial wound infection on the day 9 swab, healed later, and was the site of persistent pain continuing after wound closure. TBSA: total body surface area; nmMRSA: non-multiresistant methicillin-resistant *Staphylococcus aureus*. Image credit: Created by the authors using Matplotlib 3.10.9 (Matplotlib development team, NumFOCUS, Austin, USA).

## Discussion

This case offers a within-patient comparison of a single-application biosynthetic dressing against a conventional silver-based regimen in near-symmetrical, simultaneously acquired burns. Such a comparison controls for systemic determinants of healing that confound between-patient studies, including age, diabetes, nutrition, perfusion, medication, and adherence. It does not control for local wound factors. Here, the limbs were closely matched. Both were circumferential and predominantly mid-dermal to a similar extent. The only difference was a small deep-dermal focus of about 0.5% TBSA at the right malleoli and foot. That focus is unlikely to explain the findings. The slower healing and, above all, the persistent pain involved the whole right limb rather than the deeper areas alone. Residual confounding from it could be excluded and was the principal limitation. Its contribution was likely minor given the disparity in extent.

The differences were substantial. The Biobrane limb outperformed the silver-dressed limb in four domains: number of operations, wound infection, healing time, and pain. It needed one operation against three. It stayed infection-free, healed sooner, and was pain-free early. The silver-dressed limb became infected and was the site of all persistent pain, which outlasted wound closure and required ongoing pregabalin and amitriptyline. Both limbs were injured together and managed in one patient. The silver limb's repeated debridements, infection, and pain management were therefore the main drivers of a prolonged 23-day admission. Had both limbs behaved like the Biobrane side, the stay would likely have been shorter. Reduced length of stay is itself a documented benefit of Biobrane [6,7].

These findings mirror the trial data. Barret et al. found Biobrane reduced pain, analgesic requirement, healing time, and length of stay versus silver sulfadiazine [6]. Gerding et al. found wounds treated with Biobrane healed faster (10.6 versus 15.0 days) and were less painful at 24 hours (1.6 versus 3.6 on a five-point scale) [7]. The divergence in healing time also has consequences beyond the admission itself, since wounds that take longer than three weeks to close carry a substantially higher risk of hypertrophic scarring than those closing sooner [4]. The left limb closed at day 32, whereas the right still had an open malleolar wound at day 39. These advantages may extend to adults with mixed-depth burns and comorbidity, where comparative evidence is sparse [8,9].

Two further points merit comment. First, a perceived risk of infection beneath an occlusive biosynthetic dressing is among the most common reasons for avoiding Biobrane [8,11]. Here, it was the silver-dressed limb, not the Biobrane limb, that became infected. It developed a polymicrobial infection and needed systemic antibiotics, despite silver's antimicrobial rationale. The Biobrane limb stayed infection-free till healing. This fit controlled data that Biobrane did not increase infection risk on an adequately debrided wound [10]. The right limb was, however, deeper and repeatedly debrided, and greater depth with more devitalised tissue independently predisposes to infection [15,16].

Burn eschar is an avascular, protein-rich medium that both impairs the migration of host immune cells and restricts the delivery of systemically administered antimicrobial agents to the wound [15]. Gram-positive organisms colonise the burn surface within the first 48 hours, with gut, respiratory, and environmental flora, including gram-negative organisms, following after an average of five to seven days [15]. The growth of nmMRSA and *P. aeruginosa* on the day nine swab is consistent with that sequence, and the risk of invasive infection is influenced by the extent and depth of the injury as well as by the colonising flora [15]. Dressing choice therefore cannot be isolated as the cause.

Second, persistent post-burn pain is common, underrecognised, and linked to repeated nociceptive stimulation during dressing changes and debridement [14,17]. Neuropathic-like pain after burn injury follows a characteristic course, is typically exacerbated by weight-bearing and dependent positioning, persists on average for more than a year after injury, and is commonly managed with gabapentinoids [18]. This patient's pain matched that description: it limited standing to under three minutes at day 29 despite near-complete re-epithelialisation, and it required pregabalin and amitriptyline at discharge. It was confined to the repeatedly disturbed limb, which fits the idea that early closure and less wound-bed disturbance reduce the risk, though one case cannot prove causation. Nanocrystalline silver is also cytotoxic to keratinocytes and fibroblasts in vitro and its use is cautioned against over freshly epithelialising wounds [12]. In vitro work using silver released from a nanocrystalline silver dressing found the concentrations toxic to keratinocytes and fibroblasts to be similar to those toxic to bacteria [19]. Repeated silver exposure may, therefore, have contributed to the right limb's slower, more painful healing. These observations should nonetheless be set against the wider evidence for nanocrystalline silver, which compares favourably with older silver delivery systems on infection, operative burden, and length of stay [13]; the comparison here is between a single-application biosynthetic dressing and a competently applied modern silver regimen, not between a good option and a poor one.

The practical implication is that in mixed superficial and mid-dermal burns of indeterminate depth, possible deeper areas need not preclude a trial of biosynthetic coverage of the predominant mid-dermal component. The analgesic and operative-burden advantages may justify it. Cost-effectiveness analyses and adult randomised trials would be valuable. They should compare single-application biosynthetic dressings with modern silver dressings and include persistent pain as a prespecified outcome [9].

## Conclusions

In this adult with diabetes and near-symmetrical mixed-depth lower limb burns, the Biobrane-treated limb needed one operation, avoided infection, healed sooner, and was pain-free; the silver-dressed limb required two further debridements, became infected, and remained painful after wound closure. Because both limbs were burned at the same instant in the same patient and were of comparable depth and extent, systemic factors cannot explain the divergence, and dressing strategy is the most plausible remaining explanation, although a small deeper focus on the right limb and the limits of a single observation preclude firm attribution. Single-application biosynthetic coverage therefore merits consideration in adult mixed-depth burns, and persistent pain merits prespecification as an outcome in comparative trials.

## References

[1] Jeschke MG, van Baar ME, Choudhry MA, Chung KK, Gibran NS, Logsetty S (2020). Burn injury. Nat Rev Dis Primers.

[2] Monstrey S, Hoeksema H, Verbelen J, Pirayesh A, Blondeel P (2008). Assessment of burn depth and burn wound healing potential. Burns.

[3] Shupp JW, Nasabzadeh TJ, Rosenthal DS, Jordan MH, Fidler P, Jeng JC (2010). A review of the local pathophysiologic bases of burn wound progression. J Burn Care Res.

[4] Finnerty CC, Jeschke MG, Branski LK, Barret JP, Dziewulski P, Herndon DN (2016). Hypertrophic scarring: the greatest unmet challenge after burn injury. Lancet.

[5] Halim AS, Khoo TL, Mohd Yussof SJ (2010). Biologic and synthetic skin substitutes: an overview. Indian J Plast Surg.

[6] Barret JP, Dziewulski P, Ramzy PI, Wolf SE, Desai MH, Herndon DN (2000). Biobrane versus 1% silver sulfadiazine in second-degree pediatric burns. Plast Reconstr Surg.

[7] Gerding RL, Emerman CL, Effron D, Lukens T, Imbembo AL, Fratianne RB (1990). Outpatient management of partial-thickness burns: Biobrane versus 1% silver sulfadiazine. Ann Emerg Med.

[8] Whitaker IS, Prowse S, Potokar TS (2008). A critical evaluation of the use of Biobrane as a biologic skin substitute: a versatile tool for the plastic and reconstructive surgeon. Ann Plast Surg.

[9] Wasiak J, Cleland H, Campbell F, Spinks A (2013). Dressings for superficial and partial thickness burns. Cochrane Database Syst Rev.

[10] Lal S, Barrow RE, Wolf SE, Chinkes DL, Hart DW, Heggers JP, Herndon DN (2000). Biobrane improves wound healing in burned children without increased risk of infection. Shock.

[11] Greenwood JE, Clausen J, Kavanagh S (2009). Experience with biobrane: uses and caveats for success. Eplasty.

[12] Fong J, Wood F (2006). Nanocrystalline silver dressings in wound management: a review. Int J Nanomedicine.

[13] Nherera LM, Trueman P, Roberts CD, Berg L (2017). A systematic review and meta-analysis of clinical outcomes associated with nanocrystalline silver use compared to alternative silver delivery systems in the management of superficial and deep partial thickness burns. Burns.

[14] Summer GJ, Puntillo KA, Miaskowski C, Green PG, Levine JD (2007). Burn injury pain: the continuing challenge. J Pain.

[15] Church D, Elsayed S, Reid O, Winston B, Lindsay R (2006). Burn wound infections. Clin Microbiol Rev.

[16] Anyanwu JA, Cindass R (2023). Burn debridement, grafting, and reconstruction. StatPearls [Internet].

[17] Dauber A, Osgood PF, Breslau AJ, Vernon HL, Carr DB (2002). Chronic persistent pain after severe burns: a survey of 358 burn survivors. Pain Med.

[18] Schneider JC, Harris NL, El Shami A, Sheridan RL, Schulz JT 3rd, Bilodeau ML, Ryan CM (2006). A descriptive review of neuropathic-like pain after burn injury. J Burn Care Res.

[19] Poon VK, Burd A (2004). In vitro cytotoxity of silver: implication for clinical wound care. Burns.

